# Exploring LGBTQ+ youth well-being: Utilizing a mixed-method approach to uncover insights, needs, and strategies of LGBTQ+ youth in Northwest Arkansas

**DOI:** 10.1371/journal.pone.0316885

**Published:** 2025-01-03

**Authors:** Cristobal Calvillo, Camille Richoux, S. Alexandra Marshall

**Affiliations:** Department of Health Behavior and Health Education, Fay W. Boozman College of Public Health, University of Arkansas for Medical Sciences, Little Rock, Arkansas, United States of America; Far Eastern University - Manila, PHILIPPINES

## Abstract

**Objective:**

This study investigates the well-being and needs of LGBTQ+ youth in Northwest Arkansas, aiming to understand factors influencing their quality of life and inform supportive policies and practices.

**Methods:**

This exploratory, descriptive evaluation used a sequential explanatory mixed methods design to explore LGBTQ+ youth well-being and needs in Northwest Arkansas. 218 online survey respondents and six interviewees under 21 who self-identified as LGBTQ+ participated. Adult stakeholders (n = 16) also completed complementary interviews providing their perceptions of the needs and well-being of LGBTQ+ youth in the area.

**Results:**

The findings highlight the crucial need for confidantes (82.5% of respondents) and a sense of safety within homes (80.3%). Notably, 51.8% sought mental health care. The qualitative interviews uncovered five themes regarding LGBTQ+ youth’s perceived quality of life, echoed in additional perspectives from adult stakeholders.

**Conclusions:**

While many of the participating youth felt safe and supported at the time of data collection, enhancing well-being for LGBTQ+ youth beyond the study’s context requires strategies such as promoting community acceptance, creating supportive spaces, and enhancing empathetic engagement among adults. Adult stakeholders were keenly aware of the roles that politics and education played along with the importance of access to resources and safe spaces. These findings support inclusive policies and programs to foster a more equitable environment in the state and beyond.

## Introduction

Individuals under 21 years old who identify as lesbian, gay, bisexual, transgender, queer, or with other diverse sexual and gender identities falling within the LGBTQ+ spectrum can be characterized as LGBTQ+ youth. LGBTQ+ individuals are increasingly recognizing and revealing their identities at younger ages within their families, schools, and communities. Recent studies have indicated a trend toward a decrease in the age at which sexual minority individuals come out, particularly during adolescence. This adolescent period is marked by significant developmental milestones, including self-awareness and the influence of social norms surrounding sexuality and gender [1–6]. However, while understanding, labelling, and sharing an LGBTQ+ identity during adolescence can have developmental advantages, it also exposes young individuals to heightened vulnerability [7, 8].

### Situation of LGBTQ+ youth in the United States

In the United States context, LGBTQ+ youth ages 13–17 are currently estimated to make up 9.5% of the population of youth. LGBTQ+ individuals have unique health needs, and many experience numerous health disparities. (Note: Health disparities are preventable differences experienced by socially disadvantaged populations; they are ‘inequitable and are directly related to the historical and current unequal distribution of social, political, economic, and environmental resources’ [9].) Further, while many LGBTQ+ youth can thrive during adolescence, others experience bullying, discrimination, and stigma, all of which can contribute to negative health outcomes [9]. Also, recent research published by the Trevor Project indicates that suicidal thoughts among LGBTQ+ youth have trended upward, but youth who felt supported at home, at school, or in their communities had significantly fewer suicidal attempts [10]. Other research has concluded that social support, for LGBTQ+ youth, positively impacts health outcomes and vice versa. Low social support relates to increased adverse mental and behavioural outcomes, including depression, anxiety, suicide, drug and alcohol use, and poor sexual health [11]. The adverse experiences that result in these outcomes appear to be experienced more frequently by LGBTQ+ youth than their heterosexual and cisgender counterparts [3, 12, 13].

Within the school context, some research has shown negative outcomes for SGM youth. For example, the Human Rights Campaign’s (HRC) analysis of the CDC’s Youth Risk Behavior Survey data showed that American LGBTQ+ youth experienced bullying and felt unsafe at school more frequently compared to non-LGBTQ+ youth [14]. The meta-analytic review by Toomey and Russell [15] revealed that sexual minority youth experienced moderately higher levels of school-based victimization compared to heterosexual youth. Additionally, when LGBTQ+ youth students do report victimization, their issues are less likely to be addressed by teachers or school staff and more likely to persist compared to those of heterosexual, cisgender students [16]. Therefore, LGBTQ+ youth comprise a growing percentage of the population in the United States and have unique health/healthcare needs warranting support.

### The sociopolitical context

Arkansas is a state in the southern United States where, according to a Religious Landscape Study, most adults in Arkansas identify as Christian (79%), and more adults are registered as Republicans (41%) or identify as politically Conservative (42%) compared to other political parties or ideologies in the state [17]. These religious and political ideologies often articulate beliefs and formulate policies that create an unwelcoming, unaccepting, or even hostile environment for LGBTQ+ individuals in Arkansas. This is particularly concerning given the estimation that approximately 18,000 youth between the ages of 13 and 17 identify as LGBTQ+ in the state [18]. Within this sociopolitical context, there is a non-profit organization (NPO) in Northwest Arkansas (NWA) that aims to support LGBTQ+ youth by providing safe spaces, inclusive programming, and tailored services responsive to the needs and interests of local LGBTQ+ youth.

Therefore, due to the increasing presence of the LGBTQ+ youth population in Arkansas and in the US, the urgent mental health and healthcare needs of LGBTQ+ youth, and the prevailing sociocultural climate in the state, the NPO believed there was a need for a comprehensive assessment of the availability and accessibility of social support and healthcare services for LGBTQ+ youth in NWA. Thus, the purpose of the present evaluation study was to provide insights into the overall well-being and needs of LGBTQ+ youth in NWA. In addition, the research team aimed to identify the factors affecting the quality of life of LGBTQ+ youth in Arkansas and intends to inform policy and practice and to enhance the quality of life for LGBTQ+ youth in Arkansas.

This evaluation study utilized a mixed methods approach [19]. This approach facilitated the complementary utilization of quantitative and qualitative data to comprehensively address the insights, needs, and factors related to the quality of life of the participating LGBTQ+ youth and their stakeholder´s perceptions of the current quality of life for LGBTQ+ youth in Arkansas. The study was guided by three distinct questions: (1) How do young LGBTQ+ Arkansans experience the current quality of life? (2) What specific needs do LGBTQ+ youth in Arkansas have to achieve an improved quality of life? and (3) What are the barriers or gaps hindering the realization of these needs?

## Material and methods

### Study design

This study was an exploratory and descriptive evaluation that used an explanatory sequential mixed methods design. This design began with quantitative data collection and analysis explained through a qualitative follow-up to better understand or explain the quantitative data [19]. For the quantitative evaluation, an online questionnaire was administered, and the qualitative evaluation consisted of interviews conducted via Zoom or email. The research team quickly began “connecting data” collected by the quantitative survey to inform the subsequent collection of qualitative data collected during the interviews [20]. Recruitment and data were collected between May, 1st and September, 30th of 2022 in Arkansas, USA. The Institutional Review Board (IRB) at the University of Arkansas for Medical Sciences deemed the project (#274275) not to be human subjects research, since it did not meet the definition of “research”–being an evaluation conducted on behalf of the NPO–and determined that IRB oversight was not required. The LGBTQ+ NPO shared recruitment materials on its social media and at LGBTQ+ pride events held in NWA. Following the granting of consent, youth participants furnished demographic details, including gender identity, age, race, sexual orientation, school grade levels, and counties of residence. Subsequently, they responded to the survey questions organized in topically grouped sets of questions. In the qualitative analysis, insights garnered from interviews were categorized into several thematic areas.

### Participants

For the study’s sample, a total of 224 LGBTQ+ youth were obtained. Out of this sample, data were analysed from the 218 LGBTQ+ youth who completed the survey, along with the 6 LGBTQ+ youth and 16 adult stakeholders who completed qualitative interviews. Participants provided electronic consent or assent before completing the online survey and the interview participants provided verbal consent before completing the interview. For the participation of minors, prior consent was obtained from their parents or guardians by the NPO.

### Measures

For this evaluation study, the research team utilized two primary methods: a cross-sectional, one-time, online survey and one-time, semi-structured, individual interviews. To develop the question domains, survey items, and interview guide, the researchers partnered with an LGBTQ+ NPO to understand their organization’s objectives and purpose of the evaluation and utilized existing LGBTQ+ survey tools as guides. The survey tools referenced included the San Francisco Bay Area’s Needs Assessment [21] and the CDC’s Youth Risk Behavior Surveillance (YRBS) [22]. Advertisements about the study, which included a link to the online survey, were distributed electronically to contacts of the LGBTQ+ NPO via social media posts, radio, and emails. The LGBTQ+ NPO also directly connected with school-based Genders and Sexualities Alliances (GSAs)to promote the survey to students during their regular meetings. The NPO also distributed information about the study at LGBTQ+ Pride events held during the month of June 2022.

For the one-time, semi-structured individual interviews. All participants were recruited by the NPO using their existing contacts, and participants consented to complete the interview. All interviews were conducted by a member of the research team who self-identified as a member of the LGBTQ+ community and had a rapport with the NPO and its clients and stakeholders. The interviews were either completed via Zoom [23] or email since previous research has found no effect on data quality when comparing face-to-face and electronic data collection methods among youth [24]. Interviews that were completed via Zoom [23] were recorded, and the audio recordings were professionally transcribed for analysis. Participants completing their interview via email typed their responses to the questions onto a Word document and emailed the documents back to the research assistant, and those completed documents served as the transcripts for analysis.

### Analysis

For quantitative data, an online questionnaire was created on the Google Forms platform. Then, after the data was collected, it was downloaded into an Excel file and then uploaded and analysed in SAS (SAS, Version 9.4 TS Level 1M7). Data were removed and excluded from the quantitative analysis if the survey respondent met one or more of the following criteria for exclusion: (1) selected all available response options; (2) indicated reporting from locations other than Northwest Arkansas; (3) exceeded the desired age range; (4) duplicated responses; and (5) responded to less than 70% of the survey questions. The final dataset (N = 218) was analysed using descriptive statistics. This allowed us to summarize key variables, highlighting central tendencies, and distributions. By focusing on frequencies, we gained a clear understanding of the data’s core characteristics.

For the qualitative information, the data from the interview transcripts were organized and coded using MAXQDA22 qualitative data analysis software [25]. Two members of the research team used conventional methods for content analysis [26]. The research team used two coders to enhance the rigor of the analysis [27]. The researchers coded the transcripts separately following two codebooks–one for youth and one for stakeholders–that were developed and agreed upon with consensus. The researchers met after an initial round of coding to compare coding schema, discuss discrepancies in applications of codes, and, finally, achieve consensus in coding [26, 27]. Then, they met to review the coding and generate themes.

## Results

### Survey data from LGBTQ+ youth

Regarding the sociodemographic characteristics of the 218 LGBTQ+ youth participants who completed the survey, Table 1 provides information about gender identity. Among those who identified as cisgender (n = 89), the majority identified as cisgender females (77.5%). Among those who identified as transgender (n = 129) many identified as non-binary (59.7%). Table 2 provides data on age, race, sexual orientation, grade in school, and county of residence in the state. Many participants were 16 years old (25.7%) or 15 years old (22.9%). Most participants identified as Caucasian (64.6%), followed by those who identified as bi- or multiracial (10.65%), and then African American and Latinx participants, both at 8.2%. Concerning sexual orientation, nearly half of the sample reported being either bisexual or pansexual (45.9%). Regarding school grades, the tenth grade was the most frequently selected grade level (29%). Slightly more than half of the participants (55%) reported residing in Washington County.

**Table 1:** Gender identity (N = 218).

	Gender Non-binary^1^	Gender Male^2^	Gender Female^3^	*Category total*
**Gender identity**	***n* (%)**	***n* (%)**	***n* (%)**	***(n)***
Transgender	77 (59.7)	43 (33.3)	9 (7)	129
Cisgender	0 (0)	20 (22.5)	69 (77.5)	89

Note.

^1^Subjects who indicated some identity(ies) within the non-binary trans response options, such as non-binary, gender-fluid, and/or gender queer.

^2^Subjects who indicated solely or primarily male identity within the response options.

^3^Subjects who indicated solely or primarily female identity within the response options.

**Table 2:** Sociodemographic variables (N = 218).

**Age (years)**	***n* (%)**
13	18 (8.3)
14	13 (6)
15	50 (22.9)
16	56 (25.7)
17	38 (17.4)
18	24 (11)
19	13 (6)
20	4 (1.8)
21	2 (0.9)
**Age rank**	**Min-max**
	13–21
**Age mean**	***M***
	16.10
**Race**	***n* (%)**
Bi- or Multi- Racial^4^	23 (10.6)
Black‎/African American	18 (8.2)
East Asian (Chinese)	1 (0.5)
Latina‎/o‎/Latinx	18 (8.2)
Middle Eastern	1 (0.5)
Native American‎/American Indian or Alaska Native	13 (6)
Pacific Islander, including Native Hawaiian	3 (1.4)
White‎/Caucasian	141 (64.6)
**Sexual orientation**	***n* (%)**
Gay	22 (10.1)
Bisexual or Pansexual	100 (45.9)
Lesbian	52 (23.8)
Others	44 (20.2)
**School grade levels**	***n* (%)**
6th	2 (0.9)
7th	9 (4.1)
8th	16 (7.3)
9th	27 (12.5)
10th	65 (29.8)
11th	46 (21.1)
12th	34 (15.6)
Homeschooled	16 (7.3)
Not in school	3 (1.4)
**Counties of residence**	***n* (%)**
Arkansas	2 (0.9)
Benton	87 (40.2)
Carrol	1 (0.5)
Crawford	1 (0.5)
Faulkner	1 (0.5)
Franklin	1 (0.5)
Independence	2 (0.9)
Marion	1 (0.5)
Sebastian	1 (0.5)
Washington	120 (55)

Note

^4^Subjects who indicated more than one option in relation to the Race response items.

Regarding the needs assessment, the results in Table 3 show that the most frequently reported personal aspect of being an LGBTQ+ identified youth was having someone to confide in (82.5%). This was followed by having support from friends or classmates at school (77.5%) and, thirdly, having a sense of connection to other LGBTQ+ community members (75.2%).

**Table 3:** Frequency in different personal aspects in LGBTQ+ youth (N = 218).

Personal aspects in LGBTQ+ youth	Not interested	Disagree	Agree
	*n (%)*	*n (%)*	*n (%)*
I am comfortable being as openly out as I want to be most of the time in the city/town where I live	2 (0.9)	72 (33)	144 (66.1)
I have someone to confide In or talk about my problems	3 (1.4)	35 (16.1)	180 (82.5)
I have a sense of connection with my other LGBTQ+ community members where I live	3 (1.4)	51 (23.4)	164 (75.2)
I have experienced bullying/harassment from students at school regarding my orientation/gender identity	16 (7.3)	70 (32.1)	132 (60.6)
I have experienced bullying/harassment from faculty or staff at school regarding my orientation/gender identity	17 (7.8)	104 (47.7)	97 (44.5)
I have received support from school faculty or staff to help with bullying/harassment	51 (23.4)	70 (32.1)	97 (44.5)
I have received support from friends and classmates at school to help with bullying/harassment	42 (19.3)	35 (16.1)	141 (64.6)
I have received support from school faculty or staff in relation to my sexual orientation or gender identity	30 (13.8)	73 (33.5)	115 (52.7)
I have received support from friends or classmates at school in relation to my sexual orientation or gender identity	15 (6.9)	34 (15.6)	169 (77.5)
I have received support from family at home in relation to my sexual orientation or gender identity	8 (3.7)	77 (35.3)	133 (61)
I have received support from extended family in relation to my sexual orientation or gender identity	30 (13.8)	107 (49)	81 (37.2)

In terms of safety, Table 4 shows that many youth respondents felt safe at home (80.3%), at a public library (77.1%), or at a local business or coffee shop (67.5%). Conversely, the places where they reported feeling less safe included around the police (54.1%), at a place of worship (45.4%), and work (42.2%).

**Table 4:** Frequency of the degree of unsafety and safety in different contexts in LGBTQ+ youth. (N = 218).

Context in which I feel safe	Not applicable or do not know	Unsafe	Safe
	*n (%)*	*n (%)*	*n (%)*
Safe at home	9 (4.1)	34 (15.6)	175 (80.3)
Safe at school	8 (3.7)	66 (30.3)	144 (66)
Safe at place of worship	73 (33.5)	99 (45.4)	46 (21.1)
Safe in neighborhood	30 (13.8)	73 (33.5)	115 (52.7)
Safe in town or city	15 (6.9)	75 (34.4)	128 (58.7)
Safe in a relationship or dating	51 (23.4)	33 (15.1)	134 (61.5)
Safe in using public transportation	56 (25.7)	86 (39.4)	76 (34.9)
Safe around police	24 (11)	118 (54.1)	76 (34.9)
Safe at work	92 (42.2)	33 (15.1)	93 (42.7)
Safe around a doctor or health professional	21 (9.6)	59 (27.1)	138 (63.3)
Safe at large events, concerts, or festivals	27 (12.4)	64 (29.4)	127 (58.2)
Safe at local businesses or coffee shops	16 (7.3)	55 (25.2)	147 (67.5)
Safe at a public library	21 (9.6)	29 (13.3)	168 (77.1)

In addition, from Table 5, the events with the highest percentages of interest among LGBTQ+ youth show varied levels of engagement. In the "not interested" category, poetry slams or open music nights (26.1%), group mental health support (21.6%), and clothing swaps (18.8%) had the highest percentages, indicating less appeal for these activities. For the "sort of interested/indifferent" category, group mental health support (46.3%), book clubs (39%), and poetry slams or open music nights (37.7%) garnered the most indifference. Conversely, in the "very interested" category, drop-in safe spaces to hang out with friends or other LGBTQ+ peers (79.4%), game nights (67.4%), and affirming parties, dances, or proms (66.5%) were the most popular, reflecting a strong preference for inclusive and social environments where LGBTQ+ youth can feel accepted and engaged.

**Table 5:** Frequency of interest in participating in several events for LGBTQ+ youth. (N = 218).

How interested would you be in participating in each of these?	Not interested	Sort of interested / indifferent	Very interested
	*n (%)*	*n (%)*	*n (%)*
LGBTQ+ family events (cookouts, potlucks)	14 (6.4)	77 (35.3)	127 (58.3)
School-based LGBTQ+ support groups	18 (8.3)	57 (26.1)	143 (65.6)
Mental health support (individual)	34 (15.6)	76 (34.9)	108 (49.5)
Mental health support (group)	47 (21.6)	101 (46.3)	70 (32.1)
Festivals	17 (7.8)	66 (30.3)	135 (61.9)
Clothing swaps	41 (18.8)	71 (32.6)	106 (48.6)
Affirming parties, dances, or proms	22 (10.1)	51 (23.4)	145 (66.5)
Game nights	23 (10.6)	48 (22)	147 (67.4)
Book clubs	41 (18.8)	85 (39)	92 (42.2)
Art and craft events	37 (17)	55 (25.2)	126 (57.8)
Poetry slams or open music nights	57 (26.1)	82 (37.7)	79 (36.2)
Drop-in safe spaces to hang out with friends or other LGBTIQ+ peers	11 (5)	34 (15.6)	173 (79.4)
LGBTIQ+ mentoring or tutoring	26 (11.9)	76 (34.9)	116 (53.2)

Regarding healthcare, 51.8% (n = 113) of the youth had visited a mental health provider, while 24.8% had not but expressed a need for one, and 23.4% had not visited and did not need one. Among those who had visited (n = 113), 77.9% (n = 88) did not feel that their mental health provider was sensitive to their needs or identity as an LGBTQ+ person. Additionally, among the subset of transgender youth (n = 129), 50.4% had not visited a gender-affirming healthcare provider but did not need one; whereas 31.8% had not visited but did need one, and 17.8% had visited a gender-affirming healthcare provider. Various reasons were reported for not visiting a mental health or gender care provider. Table 6 highlights the most prevalent concerns, which included worries about services lacking LGBTQ+ friendliness (15.8%), fear of someone discovering their use of these services (14.5%), and apprehension regarding disclosure to their parents or guardians (13.4%).

**Table 6:** Frequency of the reason(s) for not visiting a gender care or mental health provider (n = 129)*.

If you did not visit a gender care or mental health provider in the past year but needed to, what was the reason?	*n (%)*
*Participants could select more than one response or not select any response options for these items.	
Concerned the services would not be LGBTQ+ friendly	73 (15.8%)
Being afraid someone would find out I was using this service	67 (14.5%)
Being afraid that my parents/guardians would be notified	62 (13.4%)
Not able to afford services	55 (11.9%)
Did not know where to access this service	52 (11.3%)
Concerned the services would not be for people of my age	48 (10.4%)
Being afraid that my parents/guardians would get in trouble for getting me these services (reported to Child Protective Services)	40 (8.7%)
Not able to get transportation and/or too far away from me	30 (6.7%)
Not having someone to take care of other family member so I could go	14 (3%)
Concerned the services would not be culturally sensitive to me	13 (2.8%)
Being afraid that me or my family would be reported to immigration authorities	4 (0.9%)
The services were not available in my primary language	2 (0.4%)
The services were not accessible for people with disabilities	1 (0.2%)
**Total responses**	461

### Interview data from LGBTQ+ youth

Six LGBTQ+ youth completed individual interviews via Zoom (*n* = 2) or email (*n* = 4). The oldest interviewee was 20 years old; one was 18 years old, and four were 16 years old. Most of the interviewees were white. Regarding their gender, three individuals identified as transgender; two identified as cisgender; and one did not report their gender. Most reported living in Washington County, Arkansas. Table 7 shows additional demographic characteristics of the interviewees.

**Table 7:** Demographic characteristics of LGBTQ+ youth interviewees.

Pseudonym	Age	Education level	Race/Ethnicity	Gender	Orientation	Pronouns	County	Interview method
Edwin	16	11^th^ grade	White	Trans male	Androsexual	He/him	Washington	Email
Jeremy	16	11^th^ grade	Biracial Latina	Female	Not reported	She/they	Washington	Email
Makayla	16	11^th^ grade	White	Female	Not reported	She/they	Washington	Email
Sarah	20	Not in school	White	Not reported	Pansexual	Not specified	Benton	Email
Regine	16	11^th^ grade	White	Trans female	Neptunic^5^	She/her	Washington	Zoom
Richard	18	College	White	Non-binary/Trans masculine	Bisexual	He/they	Washington	Zoom

*Note*: ^5^Refers to being attracted only to neutral non-binary, feminine non-binary, and female gender [28].

To understand the perceived quality of life among LGBTQ+ youth, the researchers examined several codes, including “sources of support,” “self-concept,” “school safety,” “home safety,” “community safety” and others. The following abbreviated themes capture their perceived quality of life and the most important factors that affect their quality of life: (1) Diverse support system; (2) importance of schools and libraries; and (3) resilience and positive self-perception. For instance, many participants mentioned family and friends as sources of support; one identified a teacher; and one mentioned their cats. Many participants identified schools, particularly Genders and Sexualities Alliances (GSAs), and libraries as safe spaces for them. All the interviewed youth reported a positive self-concept even though a few reported that they currently or previously felt lonely, sad, or depressed. Edwin said, “I feel awesome about being LGBTQ+.” However, Jeremy shared a more nuanced perspective, “I’m happy to be out to my friends, but I feel lonely and sad that my family doesn’t [accept me] and I feel like I can’t talk to them anymore.”

In addition, to assess what may be needed to improve their quality of life, the researchers pulled from the interview segments coded “care experience” as well as “wellness impact,” “gender care,” and “mental health access.” From these codes, the following abbreviated theme emerged: (4) Mixed healthcare experiences. For instance, when asked if being an LGBTQ+ identified person impacted if and when they went to the doctor, one participant said, “Yes and no, I only went if I had no other choice and the reason was because most of my doctors treated me like I was doing something wrong,” (Sarah). This differed from another participant (Regine) who indicated that she was able to get the mental health care she needed but had not pursued other care. Yet a third participant (Richard) said, “No I don’t think so,” when asked if being an LGBTQ+ person impacted his going to the doctor when needed. Lastly, to improve their quality of life in Arkansas, the youth offered some recommendations and suggested some events. (5) They want to see NPOs provide community education and outreach as well as affirming spaces (including inclusive bathrooms); specifically, they would like organizations focused on LGBTQ+ youth to provide social events, drop-in events, game nights, and support groups, and they suggest organizations improve their social media outreach. Table 8 provides a succinct list of overarching themes from the interviews with LGBTQ+ youth.

**Table 8:** Themes identified from interviews with LGBTQ+ youth.

List of overarching themes
1. The youth present a range of experiences and various sources of support that facilitate their sense of well-being.
2. Schools and libraries, in particular, were very important and provided spaces of safety for the youth.
3. Many interviewees had a positive sense of self and appeared rather resilient.
4. Some interviewees did not see a doctor or therapist, but some did, and among those who did, many reported positive experiences, but a few had negative experiences.
5. LGBTQ+ youth recommended community education and outreach as well as affirming spaces and events to improve their quality of life.

### Interview data from adult stakeholders

Sixteen adults, who interact with LGBTQ+ youth in some professional capacity and are considered stakeholders in the efforts of the LGBTQ+ NPO, completed interviews. The various professional roles held by these interview participants included a mental health care provider, a gender-affirming health care provider, an art teacher, a librarian, a parent of an LGBTQ+ youth, and representatives of other NPOs.

To address the perceptions of the current quality of life for LGBTQ+ youth in Arkansas, the researchers examined the following codes: “impressions of community” and “legal/political effects.” From these codes, the following abbreviated themes emerged: (1) Limited access to support for LGBTQ+ youth; (2) challenges in finding acceptance, especially in certain groups; (3) existence of boundaries around safe spaces; and (4) hostile political environment and negative health outcomes. Moreover, to address what LGBTQ+ youth in Arkansas need to achieve an improved quality of life, there were some substantial factors identified that need to be addressed, such as overcoming issues in the sociocultural climate and providing ample sources of support to LGBTQ+ Arkansans. Specifically, the researchers generated themes from the codes: “challenges” and “successes”. This process resulted in the following abbreviated themes: (5) External issues requiring attention, and (6) stakeholders as trusted sources of support and safe space creators. Table 9 provides a list of overarching themes from the stakeholders.

**Table 9:** Themes identified from interviews with stakeholders.

List of overarching themes
1. Many LGBTQ+ youth in Arkansas have or are able to access support but it is also limited to certain people and/or spaces.
2. Identifying support and/or feeling accepted is thought to be more difficult among certain groups (i.e., religious groups or culturally conservative communities).
3. There are boundaries–either real or perceived–around safe spaces or safe communities.
4. Hostile political environment and negative health outcomes. The political environment is hostile to LGBTQ+ youth in Arkansas causing youth to have poor mental and physical health outcomes (e.g., depression, anxiety, suicidal ideation, and self-harm).
5. There were various issues that needed to be addressed that the stakeholders viewed as external to themselves.
6. The stakeholders believed themselves to be trusted sources of support or to have successfully created a safe space for LGBTQ+ youth in their professional environments.
7. Stakeholders requested (for themselves) and recommended (for others) to have more training and resources about how to best affirm LGBTQ+ youth.

Overall, the stakeholders’ perceptions of the quality of life of LGBTQ+ youth in Arkansas were a “mixed bag”. This shared perspective indicates an awareness of the small pockets of support available to LGBTQ+ Arkansans within the broader communities that tend to be unfavourable or even hostile to LGBTQ+ individuals. Additionally, while the stakeholders believed themselves to be a source of support or safety for LGBTQ+ youth, they also believed “others,” who were either within or outside their profession, to be treating LGBTQ+ youth poorly or creating hostile environments. These “others” were some of the external factors–because the stakeholders believed them to be beyond their personal senses of agency–and were explicitly mentioned as colleagues who were openly transphobic, particular schools or communities that were unwelcoming, or locker rooms or bathrooms that were not welcoming or inclusive to gender non-conforming youth. These responses provided insight into factors that need to be addressed to improve the quality of life of LGBTQ+ youth in Arkansas.

Furthermore, the stakeholders offered some recommendations for strategies that may help improve the quality of life for LGBTQ+ Arkansans. These recommended strategies are compiled from examining the interview segments coded as “workplace development suggestions.” (7) They requested (for themselves) and recommended (for others) to have more training and resources. They also request and recommend LGBTQ+ cultural competency training, allyship training, and training on gender-affirming practices. Further suggestions are to provide resources that address referrals for affirming services and affirming groups/organizations as well as resources (or information sheets) for parents of LGBTQ+ youth and for other community members.

In general, all the stakeholders felt reasonably competent even though they had not had much–if any–formal training on affirming practices to best interact with LGBTQ+ youth, but most–if not all–recognized the need for accurate and appropriate training and believed training could be beneficial to themselves and others. Many had sought out training on their own as culturally competent training was not currently a standard part of their professional development for any of them. In addition, the stakeholders mentioned using visual cues to suggest they are affirming and trying to create a safe space for LGBTQ+ youth. Several suggested other stakeholders, or simply any adult who interacts with youth, to do the same.

## Discussion

### Aspects related to LGBTQ+ youth

The quantitative survey results and the qualitative interview findings complement each other and help provide insight into the experiences, wants, and needs of LGBTQ+ youth in Northwest Arkansas. For the most part, survey respondents indicated they felt safe and/or had sources of support. It is important to note that several of the youth, who participated in the study, were students in schools that had established Genders and Sexualities Alliance (GSA) organizations at the time of data collection, indicating some school-level support of LGBTQ+ students. Since the completion of this study, most–if not all–of those organizations have been disbanded due to new policies and legislation implemented in the state which took effect in the fall of 2022 or early in 2023. These study results may partially reflect that unique environment compared to other areas of the state where GSAs have never been in place.

Moreover, most of the youth were very interested in having drop-in safe spaces to hang out with friends or LGBTQ+ peers, game nights, or affirming parties, dances, or proms. These activities would provide additional safe spaces and opportunities for youth to build additional supportive relationships–further enhancing their overall health and well-being–and, in turn, provide a buffer from the potentially hostile climate surrounding them. As per Johnson’s research [29], these specific activities tailored for LGBTQ+ individuals are commonly referred to as “group enclosure.” These unique spaces foster a sense of safety and belonging among LGBTQ+ youth, alleviating concerns about potential mistreatment based on their sexual orientation or the need to conceal their true selves–a challenge frequently encountered in public settings [30, 31]. Furthermore, the findings of a study conducted by Held [32] underline that LGBTQ+ individuals exhibit increased feelings of security and comfort in LGBTQ+-only environments when compared to conventional spaces. Finally, these dedicated LGBTQ+ leisure spaces can be a place where young individuals empower themselves to engage freely in activities that do not follow traditional gender roles and enable genuine self-expression [31]. Still, the potential benefits of such activities cannot be overstated.

Social support, fostering inclusive and affirming environments, providing positive role models, and developing coping strategies have all been identified in previous research as protective factors for LGBTQ+ youth [10, 33]. Expanding on this, Hall’s systematic review [34] delves into the protective elements for LGBTQ+ young individuals. Notably, support from friends who also identify as LGBTQ+ can be particularly beneficial. Such support provides a comfortable space for discussing LGBTQ+ issues, fosters a sense of shared experience, offers guidance on navigating challenging situations (such as difficult family reactions), and introduces positive role models who are content and at ease with their LGBTQ+ identity [35]. Regarding the matter of inclusive environments, a recent study conducted by Wilson and Cariola [36] provides evidence that such environments, in conjunction with antidiscrimination policies, serve to mitigate the challenges faced by sexual and gender minority youth in comparison to their counterparts. If NPOs offer the types of activities mentioned, these can also help foster the resilience of LGBTQ+ youth. Since some of the youth interviewees already appeared resilient and were engaged with a NPO, one could conclude that the organization is already providing activities that foster resilience and should continue to do more of what they are already doing. Nonetheless, it would be beneficial to implement more activities mentioned specifically by the youth, and in doing so, the NPO may experience increased participation and improved engagement by the youth.

### Aspects related to adult stakeholders

Interviews by adult stakeholders presented valuable perspectives to consider. Their perceptions of the quality of life of LGBTQ+ youth in Arkansas were likely very realistic. Speaking from their professional interactions with LGBTQ+ youth, some stakeholders shared their awareness of the effects of certain policies and the sociocultural climate on adolescent lives, and some spoke from their own lived experiences on these matters. They spoke of the role they play (or have played) in the lives of LGBTQ+ youth and of an awareness of the impact that other adults can play in the lives of LGBTQ+ youth.

The instrumental role of adults can profoundly influence youth outcomes, serving as risk or protective factors [10]. Numerous research studies have shown that supportive connections with caring adults, regardless of their role (teacher, parent, relative, or adult within the community), provide a crucial protective factor for LGBTQ+ youth [36]. Research shows adult support directly and indirectly contributes to reduced depression, substance use, and school avoidance among LGBTQ+ youth [37, 38]. Additional studies reveal caring adults can protect against substance abuse [39] and suicidal ideation [40–42]. A systematic review by Johns et al. [43] found parental support associated with fewer depressive symptoms and higher self-esteem for transgender youth, while trusted adult support correlated with less school absenteeism and greater feelings of school safety. In essence, fostering supportive relationships with adults appears critical to promoting mental health and well-being in this at-risk population [44]. Moreover, adult support serves as a protective factor that can mitigate experiences of victimization [38]. Notably, parental support early in critical identity development stages exhibits heightened impacts on well-being. Cultivating strong bonds between LGBTQ+ youth and affirming adult allies lays a foundation for wellness during vulnerable developmental periods. For this reason, while the LGBTQ+ NPO may not currently create educational resources or provide training to adults who interact with LGBTQ+ youth, the researchers would encourage them to consider developing these tools in the near future. Such activities’ effects on adults’ knowledge and attitudes could be measured in future research projects.

## Limitations

Study limitations include reliance primarily on an LGBTQ+ organization’s existing contacts for recruitment, which may bias the sample towards youth who are relatively more open about their identity and have increased social support. Additionally, conducting interviews with youth still living at home introduced logistical barriers that may have inhibited candid responses due to a lack of privacy and concerns about family members being present or overhearing. Furthermore, the incorporation of quantitative psychosocial measurements in future work could enrich insights by complementing the qualitative findings on factors impacting LGBTQ+ youth well-being. Specifically, standardized assessments of aspects like social support, self-esteem, coping skills, and resilience could strengthen the mixed methods approach.

## Conclusion

The study’s findings have illuminated critical insights into supporting LGBTQ+ youth in Northwest Arkansas, leading to several important conclusions and implications for action: Firstly, despite the challenging sociocultural and political context in the state and the region of the United States, LGBTQ+ youth reported a relatively positive perceived quality of life, particularly in certain spaces and/or with select individuals. This finding suggests the importance of fostering additional community acceptance initiatives to create a more inclusive environment that extends beyond existing safe spaces. However, it’s clear from our findings that access to supportive spaces remains limited for many LGBTQ+ youth. Expanding affirming spaces, activities, and networks is imperative to provide additional avenues for support and to foster a greater sense of belonging. Peer support emerged as a fundamental need for LGBTQ+ youth from our findings, emphasizing the need to scale up opportunities for social connection. Facilitating activities that encourage peer interaction can empower identity development and bolster resilience among youth. Furthermore, our findings highlight the need for adult education and support enhancement. Many adult stakeholders recognized their limitations in providing adequate care and support and expressed a desire for education and resources. Implementing cultural competency training in various professional and community settings and offering sexuality education resources for parents and/or for professionals can equip adults with the necessary skills and knowledge to better support LGBTQ+ youth. Ultimately, by empowering adults and broadening the network of trusted individuals, we can create safer and more supportive environments for LGBTQ+ youth. These efforts have the potential to significantly enhance their mental health and overall well-being, creating a more inclusive and affirming community for all.
